# Quantitative analysis of growth and diversification in venom data using database metrics

**DOI:** 10.1093/database/baag032

**Published:** 2026-05-30

**Authors:** Kim N Kirchhoff, Tobias Senoner, Selin Tuerkoglu, Ivan Koludarov, Björn M von Reumont, Mandë Holford

**Affiliations:** Department of Organismic and Evolutionary Biology, Harvard University, 16 Divinity Avenue, 02138, Cambridge, MA, United States; Department of Informatics, Bioinformatics & Computational Biology, Technical University of Munich, Boltzmannstr. 3, 85748, Munich, Germany; Department of Informatics, Bioinformatics & Computational Biology, Technical University of Munich, Boltzmannstr. 3, 85748, Munich, Germany; Department of Informatics, Bioinformatics & Computational Biology, Technical University of Munich, Boltzmannstr. 3, 85748, Munich, Germany; Department of Entomology, Natural History Museum Karlsruhe, Karlsruhe, Erbprinzenstr. 13, 76133, Germany; Department of Organismic and Evolutionary Biology, Harvard University, 16 Divinity Avenue, 02138, Cambridge, MA, United States; Harvard Museum of Comparative Zoology, Harvard University, 26 Oxford Street, 02138, Cambridge, MA, United States; Department of Invertebrate Zoology, The American Museum of Natural History, 200 Central Park West, 10024, NYC, NY, United States

## Abstract

Animal venomics is a growing field of research with evolutionary and biotechnological significance. Yet, fundamental questions regarding the origin, diversification, and bioactivity of venoms remain unresolved. Here, we analysed venom tissue-related data curated in Tox-Prot, currently the most comprehensive database for animal venoms, across three snapshots spanning two decades (2005, 2015, 2025). We assessed the taxonomic landscape related to Tox-Prot entries, sequence length distribution, protein family abundances, and habitat-specific venom patterns. Our results consistently show that snakes, spiders, cone snails, and scorpions, along with their associated protein families such as Phospholipase A2, Snake Three Finger Toxin and Long 4(C-C) scorpion toxin family, dominate across Tox-Prot. Nevertheless, the taxonomic and protein family diversity has been steadily increasing, with 503 new species and 188 new protein families added by 2025 compared to the 2005 dataset. Marine species account for 16%–20% of total species, of which 63%–85% are Neogastropoda reflecting limited marine species diversity coverage; likewise, terrestrial taxa are disproportionately represented by Squamata (39%) and Hymenoptera (20%) relative to their natural diversities of 2.3% and 50.26%, respectively. At the molecular level, half of all entries correspond to mature peptide sequences of 26–75 amino acids, featuring three to four disulfide bridges and C-terminal amidations as the most frequently recorded post-translational modifications. Further, protein language model embeddings infer taxonomical diverse peptide and delimited large enzyme clusters. Our study maps two decades of venom data diversification, reflecting both the field’s rapid expansion and the need for robust integrated datasets to propel and disseminate that knowledge.

## Introduction

Venoms are defined as one or more bioactive compounds produced in specialized cells, tissues, or glands of an animal species. They are actively delivered into another living organism by inflicting a wound, thereby disrupting normal physiological processes for the benefit of the venomous animal [1–3]. Venoms are molecular cocktails containing from a few to over a thousand different bioactive molecules with high target specificity and potency. These bioactive compounds are mostly proteins and peptides but appear to be often accompanied by a large diversity of small molecules such as neurotransmitters, nucleosides and carbohydrates, and salts [4–6]. The range of physiological responses induced by these biomolecules spans from inflammation, pain, and bleeding to paralysis and cardiac arrest. Accordingly diverse are also the targeted receptor families and signalling pathways [4]. These properties of venom arsenals explain the successful development of 18 venom-derived therapeutics, several bioinsecticides, and various clinical markers [7, 8]. Despite this progress, little is known about the origins of venoms, the ecological triggers driving compositional and functional variability, and molecular synergisms.

**Figure 1:**
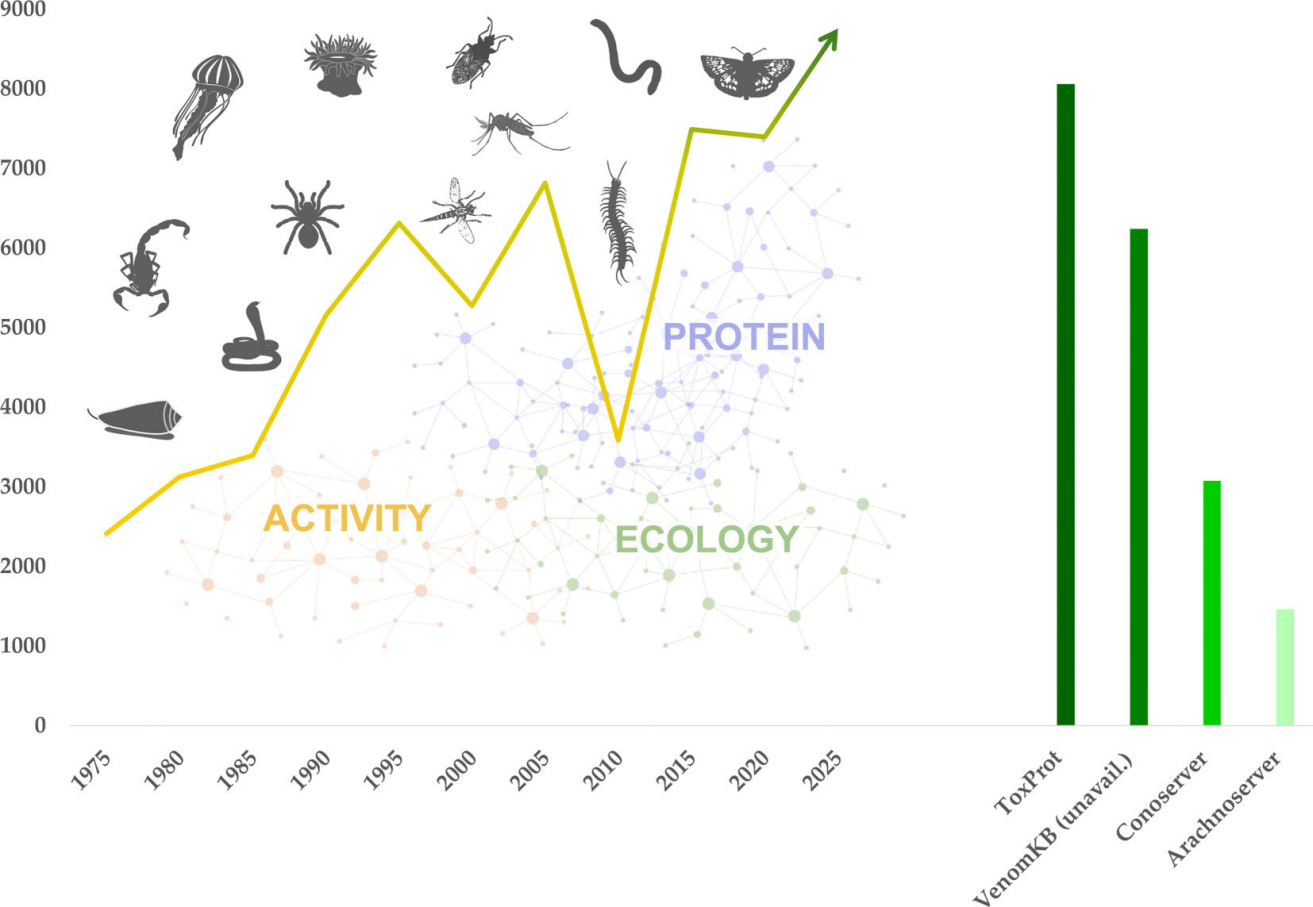
Venomics is a highly interdisciplinary research field that has grown four-fold over the last 50 years. Venoms occur throughout the animal kingdom from invertebrates such as marine snails, scorpions, and a broad diversity of insects to vertebrates such as snakes and fish. Venoms can comprise thousands of bioactive proteins and peptides that exhibit a wide array of activities often shaped by species ecology. The left chart shows the trend in published studies retrieved using the query term ‘venom’ from the PubMedCentral [11] database displayed in bins of 5 years at each point, showing a four-fold, steady increase over the last half century [11]. The associated molecular data are mostly stored in Tox-Prot [12], the most centralized venom database, but also in several smaller-scaled, taxon-specific databases such as VenomKB [52] (which is currently unreachable), ConoServer [15], and ArachnoServer [16], leading to dataset fragmentation (right chart). Animal pictograms were retrieved from www.phylopic.org.

The diversity, potency, success, and often convergent evolutionary innovation of animal venoms make modern venomics a growing field of research. This growth is further illustrated by the increasingly refined and novel platforms employed across diverse methodological approaches to study animal venoms [7, 9, 10]. The number of research articles in the PubMedCentral database [11] retrieved by the query term ‘venom’ and binned in intervals of 5 years indicates a four-fold increase over the last five decades, with intermediate peaks around the turn of the millennium and a trough around 2010 (Fig. 1; Supplementary Data S1). The data generated on venom systems encompass morphological and histological characterization of the producing organs/tissues, nucleotide and protein sequences of venom compounds, and their structural and functional properties.

Tox-Prot, an annotation project within UniProtKB, is the premier manually curated database for animal venom proteins and peptides [12, 13]. Manual curation is performed at multiple levels: (i) sequence curation, (ii) sequence analysis, (iii) literature curation, (iv) family-based curation, (v) evidence attribution, and (vi) quality assurance, integration, and updates. This process ensures that high-quality entries are included, given preference for complete sequences, protein-level verification, structural and functional information, reviewed publications, and taxonomically accurate annotations [12].

Centralized, open-source databases are indispensable for scientific research, as they curate high-quality, standardized data on a single platform, thereby fostering long-term data preservation and collaboration, supporting metadata integration, and ensuring reproducibility [9, 14]. Data centralization further enables better contextualization of findings, paving the way for hypothesis generation, comprehensive predictions, and the identification of complex relationships at the evolutionary, ecological, and molecular levels.

First launched in 2002 (with 1258 entries as of the 2004_08 release, primarily from snakes, scorpions, spiders, and cone snails) [12], Tox-Prot has since grown to include >8100 entries (release 2026_01). Nonetheless, several specialized databases, such as ConoServer [15] and ArachnoServer [16], have emerged and now host extensive data on specific taxa, contributing to dataset fragmentation and decentralization. For instance, ConoServer currently includes 3060 cone snail wild-type protein entries, whereas Tox-Prot holds 1487 Conidae entries (releases 2026_01), reflecting the complementary yet fragmented nature of venom protein resources.

In the present study, we examine entries in the central venomics database, Tox-Prot, across three time points (2005, 2015, and 2025) to assess trends in venom protein and peptide data growth, diversification, and persistent limitations. Specifically, we evaluate taxonomic diversity, protein family abundance, sequence length distributions, the representation of terrestrial vs. marine venom protein families, the prevalence of post-translational modifications (PTMs), and functional activity classifications. It must be advised that while the database is manually curated following established procedures (see the *Discussion* section) to ensure representative, high-quality, and comprehensive coverage, it does not fully capture the entirety of venom research activity. Nonetheless, by comparing these data features over time, we intend to give indicators for the field’s ongoing diversification, highlight knowledge gaps and biases, and identify areas where targeted efforts could further enhance our understanding of animal venoms.

## Materials and methods

*Data acquisition*: Toxin protein datasets were constructed from archived UniProtKB/Swiss-Prot [13] releases spanning 2005–25. For comparative analyses, three-time snapshots (2005, 2015, and 2025) were examined, while the complete 21-year timeline was used for trend analyses across venomics data. The use of archived releases ensured temporally consistent snapshots unaffected by subsequent database updates.

*Dataset extraction and filtering:* The Tox-Prot annotation project employs two complementary selection criteria to define its scope: (i) proteins with venom-related tissue expression annotations and (ii) proteins carrying the ‘Toxin’ keyword (KW-0800) in UniProt’s controlled vocabulary. These criteria are not mutually exclusive—an entry may satisfy one or both definitions.

From each Swiss-Prot release, entries matching either criterion were extracted using a custom Python parser (available at https://github.com/tsenoner/toxprot25). The parser replicates the following UniProtKB query: (taxonomy_id:33 208) AND ((cc_tissue_specificity:venom) OR (keyword:KW-0800)). Here, the taxonomy ID 33208 restricts extraction to Metazoa (animal kingdom). The tissue specificity filter (cc_tissue_specificity:venom) searches for ‘venom’ within the Tissue specificity comment (CC line) thereby capturing proteins annotated as expressed in venom glands; however, entries lacking a tissue specificity annotation are excluded—notably, entries from Cnidaria are thereby excluded. The keyword filter (keyword:KW-0800) targets entries explicitly labelled as toxins. Each extracted entry was subsequently classified according to which criterion it satisfied: venom tissue only, toxin keyword only, or both (see Fig. S1 for the overlap analysis in the 2025 dataset).

Unless otherwise noted, all analyses presented herein use the ‘venom tissue’ definition. Because the data source is Swiss-Prot (reviewed UniProtKB), all entries in the resulting datasets are expert-curated. The parser output for each annual release was saved in comma-separated value (CSV) format for downstream analysis (see https://github.com/tsenoner/toxprot25).

*Protein-family name standardization:* Protein family fields were normalized by (i) truncating at first period, comma, or semicolon to remove compound qualifiers; and (ii) expanding shorthand conotoxin codes (e.g. ‘I1 superfamily’) to full names (e.g. ‘Conotoxin I1 superfamily’). After normalization, the venom-tissue-filtered 2005, 2015, and 2025 datasets contained 76, 169, and 264 unique protein families, respectively. For cross-year visualizations, 50+ additional name mappings were applied to link renamed families across decades (e.g. ‘Snake toxin family’ to ‘Snake three-finger toxin family’).

*Habitat classification:* Each protein entry was assigned to ‘terrestrial’ or ‘marine’ habitat based on species’ known ecology. Classification used a two-tier lookup: first by taxonomic order (e.g. Scorpiones → terrestrial, Neogastropoda → marine), then by genus for orders spanning multiple habitats (e.g. within Squamata: Crotalus → terrestrial, Hydrophis → marine). The habitat-specific classification of the taxa found in our Tox-Prot dataset can be found in Supplementary Data S2.

*Taxonomic and functional annotation:* Taxonomic lineage information (Phylum, Class, Order, Family, Genus, and Species) was retrieved for each entry using the taxopy Python library, which queries the NCBI Taxonomy database [17]. Deprecated taxonomy identifiers were mapped to current NCBI IDs where necessary. Entries were grouped by taxonomic rank to identify which lineages contribute the most toxin proteins and to track newcomer taxa appearing across the decade snapshots.

*Post-translational modifications* (PTMs) were extracted from UniProtKB feature annotations, capturing disulfide bonds, glycosylation sites, modified residues, cross-links, and lipid moiety-binding regions. Modified residue descriptions (e.g. ‘4-hydroxyproline’) were mapped to standardized biological categories (e.g. ‘Hydroxylation’) using UniProt’s controlled vocabulary file (ptmlist.txt), downloaded from the UniProtKB FTP server. PTM-related keywords were additionally extracted from UniProtKB keyword annotations.

*Gene Ontology* (GO) annotations [18] were analysed across all three ontology categories: Molecular Function (MF), Biological Process (BP), and Cellular Component (CC). The GO hierarchy was parsed from the OBO format file (go-basic.obo) using the obonet Python library. Annotation coverage was calculated as the percentage of entries with at least one GO term per category, and the most frequent terms were tracked across the 21-year timeline.

Summary statistics were computed for each dataset, including total entry counts, unique protein families, fragment status, PTM annotation coverage, toxic dose annotation prevalence, and species/order diversity. Sequence length distributions were analysed upon mature protein sequences using 25 amino acid (aa) bins.

*ProtSpace:* For sequence processing, signal peptides and propeptides were removed when present, based on UniProtKB feature annotations. Entries without these annotations were assumed to already represent the mature form. Protein embeddings were generated using the ProtT5 protein language model [19]. The high-dimensional embedding space was visualized using ProtSpace [20] with UMAP [21] dimensionality reduction (n_neighbors=50, min_dist=0.5). Protein families were colour-coded using the same scheme as in Fig. 3. Clustering quality was quantified using silhouette scores [22], which measure separation between protein families in the embedding space (values range from −1 to 1; higher indicates better clustering). Five variants were compared: (1) full-length sequences including signal peptides, (2) signal peptides removed, (3) signal peptide removed excluding fragments, (4) signal peptides and propeptides removed, and (5) signal peptides and propeptides removed excluding fragments (Fig. 7; Fig. S5).

*Code availability and reproducibility:* The complete analysis pipeline, including data parsing scripts and analysis code, is publicly available at https://github.com/tsenoner/toxprot25. The repository contains source code and documentation to reproduce all data processing and analysis steps. Processed toxin datasets for all 21 years (toxprot_2005.csv through toxprot_2025.csv) are provided in the repository’s data/processed/toxprot directory.

## Results

### Trends in venomics data: comparative analysis across two decades of Tox-Prot data

**Figure 2:**
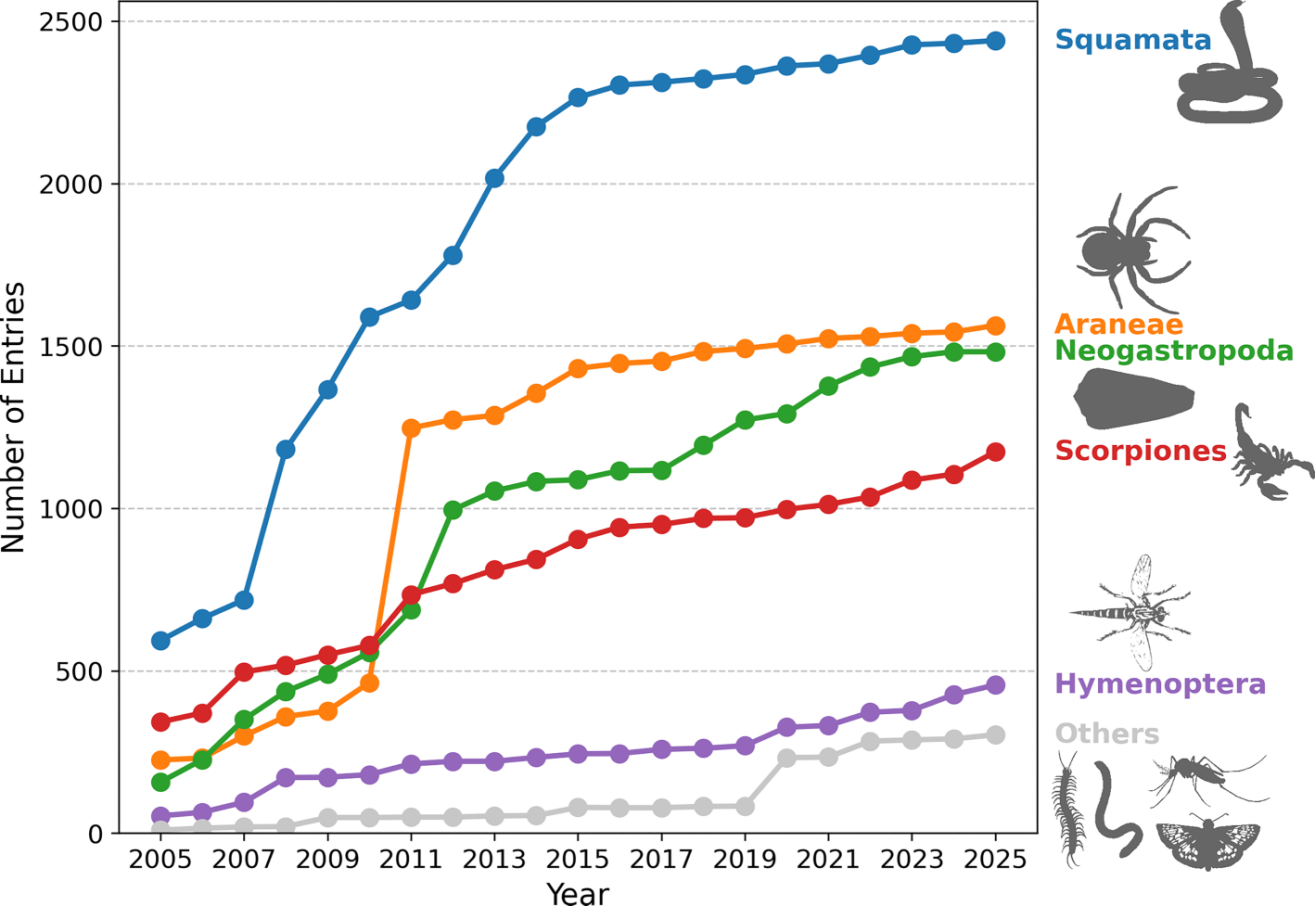
Squamata (snakes and lizards) are the most represented order in Tox-Prot, accounting for roughly a third of all venom protein entries throughout two decades of database growth. The line chart tracks the number of entries for the five most represented taxonomic orders in Tox-Prot from 2005 to 2025. Squamata dominates consistently (∼2450 entries in 2025), followed by Araneae (spiders), Neogastropoda (cone snails), Scorpiones (scorpions), and Hymenoptera (bees, wasps, and ants). All remaining orders are aggregated as ‘Others’ (grey). The rapid growth between 2009 and 2013 reflects a major expansion in annotations for spider, cone snail, and scorpion venoms. Animal silhouettes were retrieved from PhyloPic (www.phylopic.org).

We analysed the diversity of venomous animal taxa represented in Tox-Prot database entries and their abundances, as well as the prevalence of venom protein families, sequence length distributions, the proportion of entries classified as fragments, the occurrence of PTMs, and differences between terrestrial and marine venom protein families. These categories were evaluated independently for venom tissue-related entries at three time points of the study period, i.e. for the 2005_01, 2015_01, and 2025_01 Tox-Prot dataset releases, and subsequently placed in a comparative framework (for all values and categories, see Table 1 and Supplementary Data S1). Changes in taxonomy and functional annotations were dynamically explored over the complete 21-year study period (see Fig. 2 and Fig. S4).

**Table 1.** Tox-Prot data content comparative evaluation over the last two decades.^a^

	2005	2015	2025
Total entry count	1379	6012	7418
Taxonomy: Order	8	12	18
Taxonomy: Species	257	568	760
Protein families (collapsed after first ‘,’)	76	169	264
Unassigned protein famil**y**	82 (5.9%)	549 (9.1%)	503 (6.8%)
Entries classified as ‘Fragments’	129 (9.4%)	1096 (18.2%)	1235 (16.6%)
Entries with PTM annotation	1122 (81.4%)	5222 (86.9%)	6266 (84.5%)
Entries with toxic dose annotation	212 (15.4%)	476 (7.9%)	594 (8.0%)

a The percentage given refers to the relative abundance of the total count of database entries.

### Taxonomic landscape across Tox-Prot indicates preferences in study species selection

In 2005, Tox-Prot comprised 1379 entries, compared to 6012 in 2015 and 7418 in 2025, representing an initial strong database growth of 335% between 2005 and 2015, followed by a 23% increase in the last decade. These entries were attributed to 8 and 12 taxonomic orders (257 and 568 species) in 2005 and 2015, respectively, increasing to 18 orders (760 species) in 2025. Our results indicate that venom compounds from Squamata, Araneae, Neogastropoda, and Scorpiones accounted for 95.50% (2005), 94.63% (2015), and 89.77% (2025) of all entries (Fig. 2). Ten new taxonomic orders and 503 species were added to the database between 2005 and 2025, including Scolopendromorpha (centipedes), Batrachoidiformes (toadfish), and Lepidoptera (butterflies and moths) (Fig. S2). Six taxonomic orders appear at first in our 2025 dataset such as Nectiopoda (remipedes), Phyllodocida (polychaeta), and Blenniiformes (blennies). At the taxonomic family level, several new members were added, with the highest contribution by 2025 from Scolopendridae (centipedes, 196 entries), Turridae and Terebridae (sea snails, 53 and 46 entries), and Barychelidae (brushed trapdoor spiders, 47 entries) (see Supplementary Data S1). Among those families already present in the 2005 dataset, Lycosidae (wolf spiders), Formicidae (ants), Sicariidae (six-eyed spiders), and Hemiscorpiidae (scorpions) exhibited the largest relative percentage increase by 2025. While in absolute terms, considering only the number of new entries added per taxonomic family, cone snails, snakes, and scorpions experienced the largest increase. Notably are also some disappearances such as the order Anura (tree-frogs) that appeared with four entries in the 2015 dataset but was not present in the 2005 or 2025 dataset, while the order Hemiptera: Reduviidae (assassin bugs) exhibits the opposite pattern with three entries in 2005, none in 2015, and 29 entries in 2025.

Several factors limit the assessment of venom cocktails such as their accessibility, amount, and purity. These features are particularly challenging in marine animals. Thus, we further evaluated a potential bias in the taxonomic distribution at the habitat level, differentiating between terrestrial and marine species. The seven entries for the estuarine-coastal snake *Cerberus rynchops* and the two entries for the river stingray *Potamotrygon orbignyi* were included in the marine category, while the 10 entries for the freshwater snake *Pseudoferania polylepis* were grouped within terrestrial species.

Over the two decades represented in this study, we observed a four-fold increase in marine families and species included in Tox-Port, from 3 to 12 families and from 41 to 158 species. In comparison, the number of terrestrial families doubled from 32 to 67 and the number of species increased less than three-fold from 216 to 602 species (see Supplementary Data S1). Since 2015, a stable average of 20% of database entries and species are of marine origin, up from c. 15%–16% of entries and species in 2005. Notably, while the absolute number of marine species and related proteins within Tox-Prot seems to reflect natural distribution patterns (see the *Discussion* section), it must be highlighted that 82%–85% of marine species and c. 92% of related marine venom proteins listed since 2015 are attributed to the order of Neogastropoda (i.e. Conidae, Turridae, Terebridae). In 2005, this order represented only 63% of marine species and 76% of associated entries.

The above-mentioned statistics are based solely on marine proteins and peptides found in venom systems and do not include those retrieved from Swiss-Prot using the query keyword ‘toxin’ without a venom association, thereby excluding toxic compounds from Cnidaria, Nemertea, and Porifera. When contextualizing the abundance of Neogastropoda species among marine toxic and venomous species, they still contribute with ~56% to the marine species into the database since 2015 (see Supplementary Data S1).

Comparative analysis of species diversity across terrestrial venomous taxa shows similar patterns. While the natural diversities of the orders Squamata and Scorpiones across venomous terrestrial species are estimated at 2.3% and 0.88% [23], respectively, these orders account for 39% and 16% of the 602 terrestrial venomous species included 2025_Tox-Prot dataset. This translates to an ~17- to 19-fold overrepresentation of these orders in Tox-Prot. In contrast, Hymenoptera comprises half of terrestrial venomous species in nature (50.3% in [23]) yet represent only c. 19% of the terrestrial species diversity in Tox-Prot in 2025, indicating an ~2.5-fold underrepresentation. This is even more clear on the protein entries level where Squamata contribute with 41% and Hymenoptera with only 7.8% to the number of terrestrial venom protein entries in Tox-Prot in 2025.

### Phospholipase A2, Snake three-finger toxin, and Long 4(C-C) scorpion toxin family dominate the venom protein family landscape in Tox-Prot

**Figure 3:**
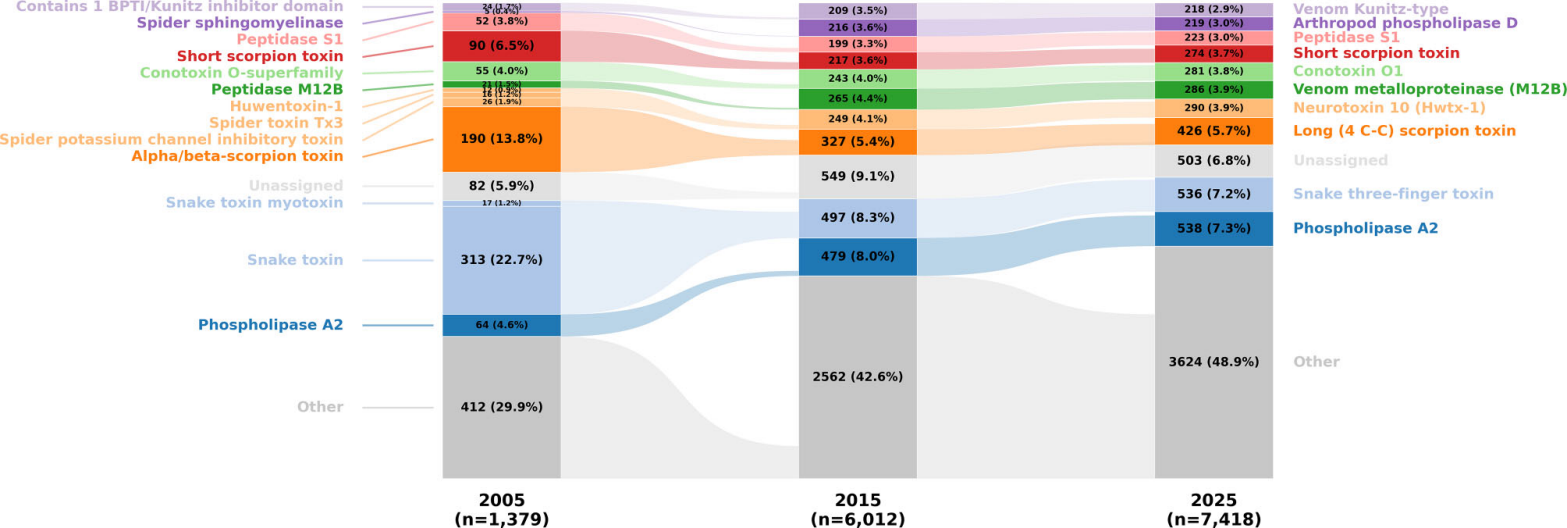
The relative abundance of the top 10 protein families in Tox-Prot remains broadly consistent over time. The alluvial plot tracks the 10 most abundant protein families (by 2025 count) in Tox-Prot in 2005, 2015, and 2025. Each bar segment represents a protein family with its absolute count and relative percentage; flows connect families across years, revealing name changes and merges (e.g. Huwentoxin-1, Spider toxin Tx3, and Spider potassium channel inhibitory toxin were consolidated into Neurotoxin 10 (Hwtx-1); Snake toxin was renamed to Snake three-finger toxin). Families are coloured by their 2025 identity, with entries lacking a family annotation grouped as ‘Unassigned’ (light grey) and all families outside the top 10 as ‘Other’ (grey). The growing ‘Other’ fraction (29.9% – 48.9%) reflects increasing taxonomic diversity in the database over time.

One hundred and eighty-eight new venom protein family names were identified in the 2025 dataset compared to the 2005 dataset. Nearly half of these new protein families were classified as Conotoxin, Neurotoxin (01–39), and Scoloptoxin (01–25), which are predominantly found in cone snails, spiders, and centipedes, respectively. However, not all of these represent newly discovered venom protein families, as most of the Neurotoxin (1–39) designations originated from renaming previously existing families, such as the Huwenotoxin-1 family, which was reclassified as Neurotoxin 10 (Hwtx-1). Despite these changes, the three most abundant venom protein families in Tox-Prot over the past two decades remained largely stable collectively comprising 20%–41% of all entries: Phospholipase A2 (PLA2), Snake three-finger toxin (3FTxs), and Long 4(C-C) scorpion toxin, with the exception that the Short scorpion toxin family was more abundant than PLA2 and ranked third in the 2005 dataset (Fig. 3).

Among the protein families present in the database throughout the complete study period (including renamed ones), particularly PLA2, Venom metalloproteinase (M12B), Peptidase S1, and Venom Kunitz-type families experienced pronounced growth, strongest during the period from 2005 to 2015 and predominantly in terrestrial taxa.

**Figure 4:**
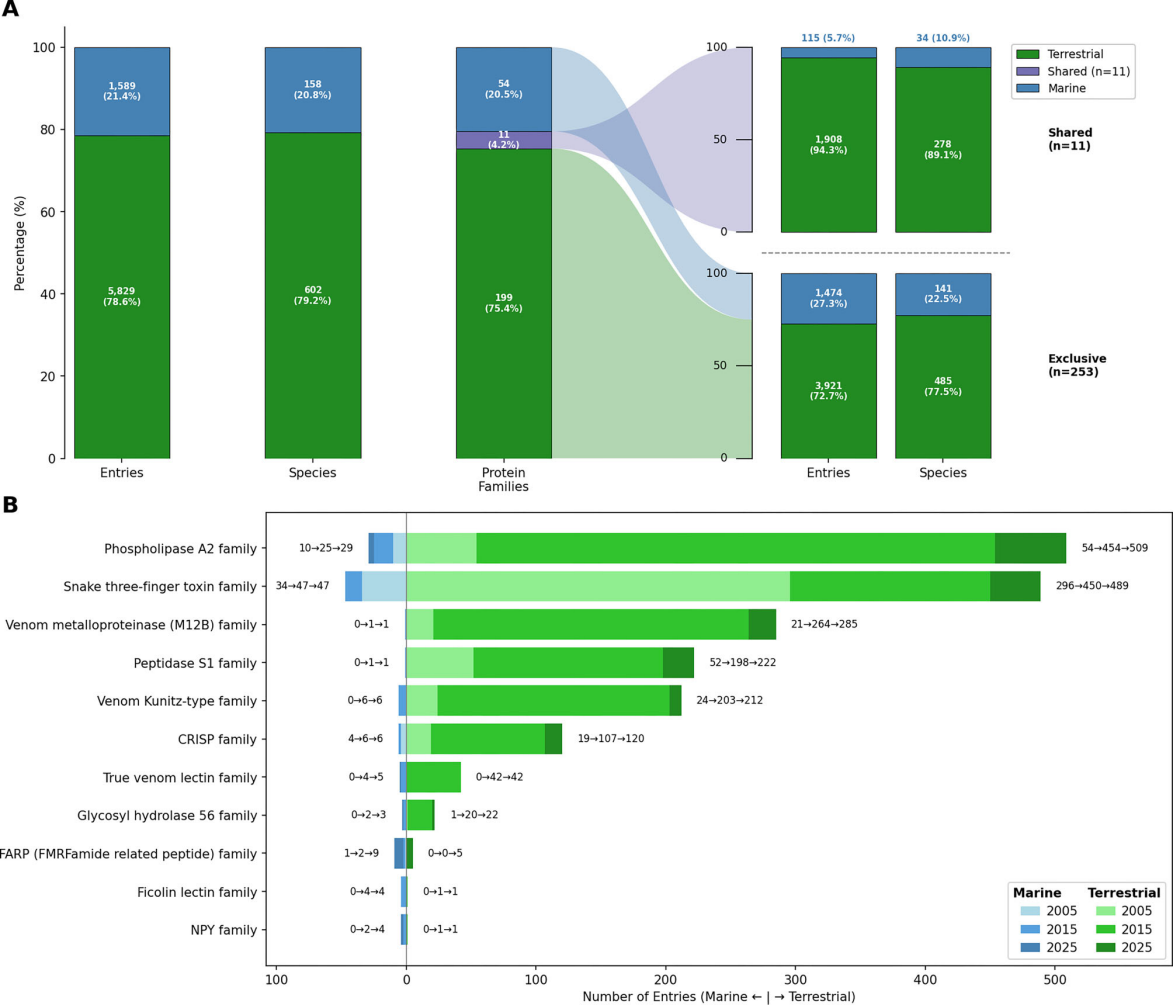
Only 11 of 264 venom protein families are shared between terrestrial and marine species, yet these families account for the majority of Tox-Prot entries. (A) Distribution of entries, species, and protein families by habitat in the 2025 Tox-Prot dataset. Terrestrial species (green, bottom) dominate in entry count, while marine species (blue, top) contribute a disproportionately large share of species diversity. The protein families bar separates into shared (purple, *n* = 11) and habitat-exclusive (*n* = 253) groups, with flows connecting to their respective breakdowns by entries and species on the right. (B) Butterfly chart of the 11 dual-habitat protein families, with marine entries extending left (blue) and terrestrial entries extending right (green). Colour shades from light to dark represent the three Tox-Prot snapshots (2005, 2015, and 2025), and annotations show the count evolution per habitat (e.g. 10→25→29). Phospholipase A2 and the Snake three-finger toxin family are the most abundant shared families. The number of shared families grew from 3 (2005) to 10 (2015) to 11 (2025), reflecting increasing cross-habitat coverage in the database.

We further analysed the distribution of venom protein families between terrestrial and marine species. Protein families uniquely associated with venom from the 11 terrestrial taxonomic orders numbered 199 in 2025, while those attributed to the 8 marine orders totalled 54 families, with Squamata being present in both habitats (Fig. 4). A small subset of shared venom protein families (3 in 2005, 10 in 2015, and 11 in 2025) was identified in both groups, often with substantial representation in one and only a few entries in the other (Fig. 4). Specifically, two venom protein families showed consistently high abundance in both terrestrial and marine taxa: 3FTx and PLA2, likely attributed to (sea) snake venom proteins.

### Small proteins dominate among venom compounds, often with protein-level evidence

**Figure 5:**
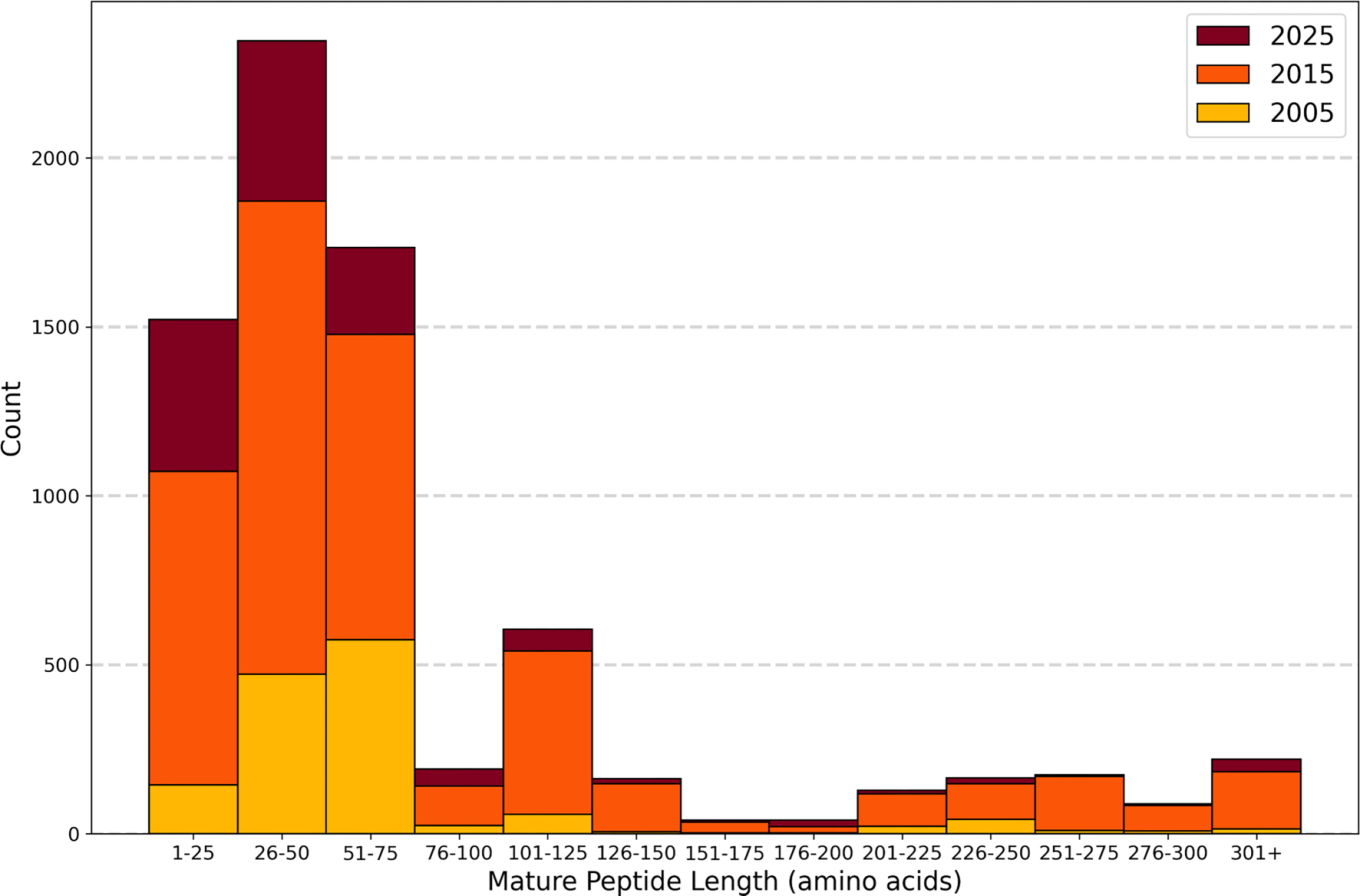
Short mature venom peptides of 26–75 amino acids (aa) dominate Tox-Prot, with the database showing a strongly right-skewed length distribution across all three time points. The stacked histogram displays the mature peptide length distribution of Tox-Prot entries, in 25 aa bins, for 2005 (yellow, bottom), 2015 (orange, middle), and 2025 (dark red, top). Mature lengths were determined using UniProtKB chain and peptide annotations where available, or by removing signal peptides and propeptides; entries without these annotations were assumed to already represent the mature form. The two most populous bins are 26–50 aa (∼2350 entries) and 51–75 aa (∼1735 entries), together accounting for >55% of all entries. Growth from 2005 to 2015 was pronounced across all size ranges, while the 2015-to-2025 increment is more modest. A secondary peak at 101–125 aa and entries >301+ likely reflects larger venom enzyme families such as metalloproteinases and phospholipases.

Mature venom peptides and proteins in the 2025 Tox-Prot dataset ranged in length from 4 to 1384 aa. Over half of all entries (55%) had a mature length between 26 and 75 aa. Between 2015 and 2025, overall database growth was 23% (Fig. 5), with the strongest growth in the shortest mature peptides (1–25 aa: 42%) and in the 176–200 aa range (91%), while longer sequences (>225 aa) showed below-average growth. Protein-level evidence was first annotated in the database in 2008, with 68.5% of the proteins having this evidence usually obtained from mass spectrometry, while the remaining were inferred from transcriptomic data. In 2025, c. 59.6% of the venom-related proteins in Tox-Prot (4424 entries) were verified at the protein level, while transcript-level evidence decreased relatively from 39.3% in 2015 to 26.2% in 2025. Proteins identified by sequence homology doubled in relative abundance to 14.1% in 2025 (Fig. S3).

Further, over the last decade, an average of 17% (i.e. 1096–1235) of database entries have been classified as ‘fragments’, compared to ∼9% (129 entries) in the 2005 dataset, which behaves in accordance to the overall database growth. Fragments are defined as proteins and peptides with at least one incomplete subsequence range, i.e. signal peptide, propeptide, or chain sequence.

### Disulfide bonds are the most prevalent post-translational modification across Tox-Prot entries

**Figure 6:**
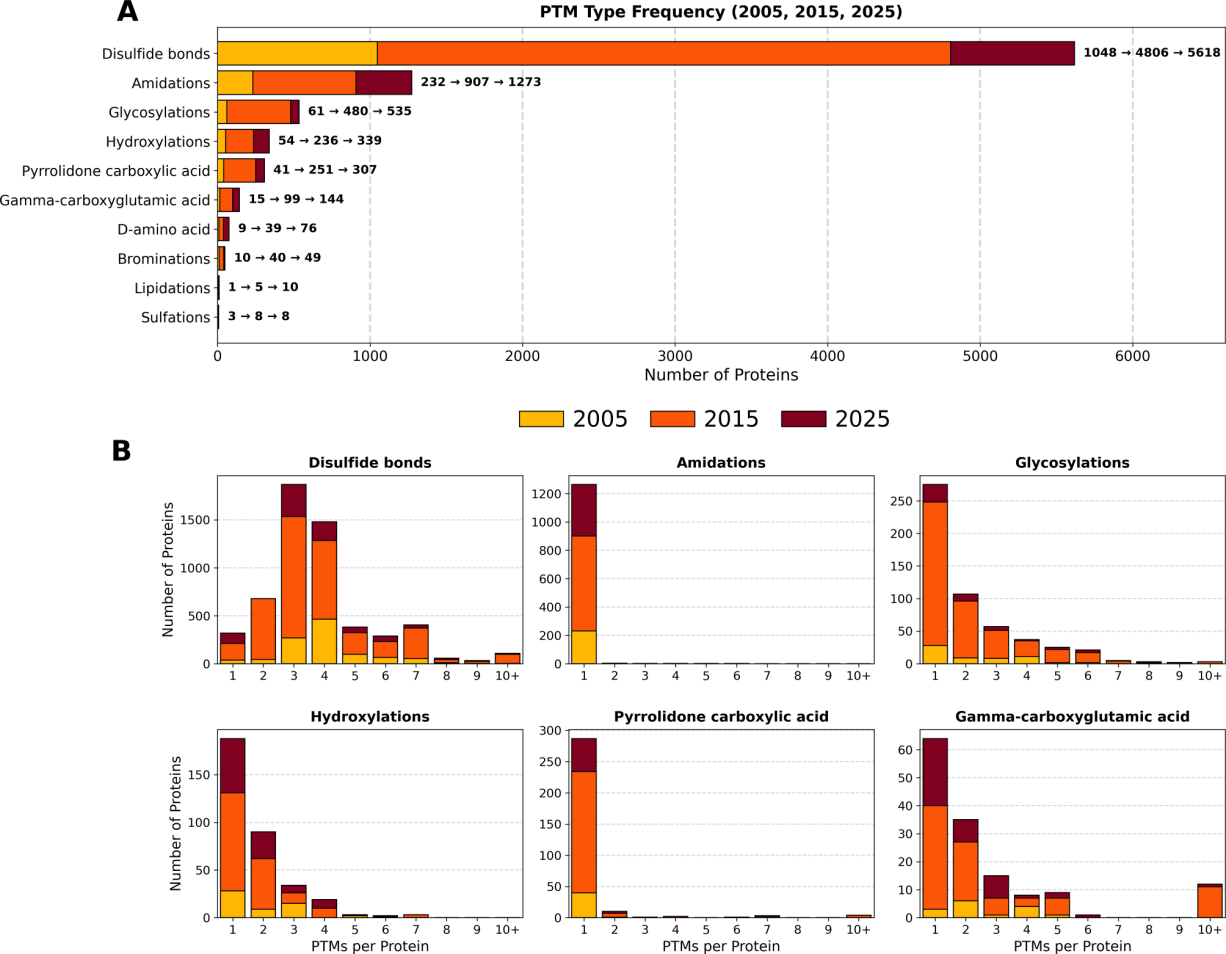
Disulfide bonds are the dominant post-translational modification in venom proteins, with three and four bridges per protein being the most frequent. (A) The 10 most common PTM types across Tox-Prot snapshots (2005, yellow-bottom; 2015, orange-middle; and 2025, dark red-top). Disulfide bonds (5618) vastly outnumber all other modifications; amidations (1273), glycosylations (535), hydroxylations (339), and pyrrolidone carboxylic acid (307) follow at considerably lower counts. Annotations show the progression of the count across the three time points (e.g. 1048→4806→5618). (B) Distribution of per-protein PTM counts for the six most common modification types. Disulfide bonds show peaks at three and four bonds (>1500 proteins each), reflecting the cysteine-rich scaffolds typical of toxin families. Most other PTMs occur predominantly once per protein.

PTMs are crucial for venom protein bioactivity, and 81%–87% of the venom proteins in Tox-Prot have carried PTM annotations since 2005. In the 2025 dataset, the most prevalent modification was the presence of three to four disulfide bonds, accounting for 53.4% of all PTM annotations, followed by amidations (20%), predominantly single amidation (Fig. 6). Other common PTMs included glycosylations, hydroxylations, pyrrolidone carboxylic acid, and gamma-carboxyglutamic acid modifications. Notably, Tox-Prot provides PTM annotations both as sequence-related features and in an additional keyword section. The number of annotated PTMs varies depending on the source used, as feature annotations provide more detailed insights into the number of each modification per protein entry. In 2025, 6266 entries were annotated with PTM sequence features, while 6932 entries were listed in the keyword section. A total of 498 entries contained annotations for both disulfide bonds and glycosylation, and 805 entries exhibit both, disulfide bonds and amidation. The venom protein families with the highest number of disulfide bond annotations included 3FTxs, PLA2, Long 4(C-C) scorpion toxin, Neurotoxin-10, and Conotoxin O1 superfamily. Amidations were also common in Long 4(C-C) scorpion toxin, Conotoxin A, and Neurotoxin-10 families, as well as in the Non-disulfide-bridged peptide superfamily and the MCD family. In contrast, glycosylation was most frequently observed in Peptidase S1, Venom metalloproteinase (M12B), Arthropod phospholipase D, Flavin monoamine oxidase, Nerve Growth Factor (NGF)-beta, and Glycosyl hydrolase 56. These patterns remained highly consistent with those observed in 2005 and 2015, with variations corresponding to database expansion and renaming/consolidation of protein families.

### Diverse functional data annotations with growing trends

The functional data annotations for venom proteins and peptides in Tox-Prot are grouped into the Function subsection and comprise, for example, free-text description about the overall protein activity, catalytic activity, biophysicochemical properties, active/binding sites, GO annotations for MF, BP, and CC categories, as well as a Keyword section with a selection of controlled vocabulary defining the proteins activity and reflecting the GO terms. Further, in the subsection for Phenotypes & Variants annotations for recorded toxic doses are given.

Starting with the latter, toxic dose data were available for 212 entries in 2005, 476 in 2015, and 594 in 2025. This data mostly comprises the lethal dose at which 50% death among test animals is reached (LD_50_), but also paralytic doses and effective doses. The individual datasets revealed that 16.5%–47.3% of venom proteins with toxic dose annotations are tested in mice (when accounting for ‘mice’ and ‘mouse’ keyword usage in the Toxic Dose category), and, on average, one-third are administrated by intravenous (i.v.) injection, followed by intraperitoneal (i.p.), subcutaneous (s.c.), and intracerebroventricular (i.c.v.) administration. Overall, the toxic dose subdataset exhibits substantial variability in test dosage units, animal frameworks, administration routes, and incubation times, which inherently limits the comparability of toxicity estimates. Nevertheless, in 2025, the most lethal venom peptides in mice included the Alpha-mammal toxins Aah2 from the Sahara scorpion and the Aagardi 3FTx from sea snake, with LD_50_ values of 0.003 and 0.005 nmol/kg, respectively, following intracerebroventricular or intravenous administration. Additionally, highly potent peptides in mice include the beta-mammal toxin Css4 from bark scorpion, delta-hexatoxin Hv1a from funnel web spider, and several textilotoxin PLA2s (homologs) from eastern brown snake, with LD_50_ values ranging between 0.012 and 0.072 nmol/kg (see Supplementary Data S1).

The evaluation of the GO annotation indicated that, initially in 2005, coverage across the three categories was very low (2%–3%), but grew to 93%–99% in MFs and CCs by 2025. However, the BP category indicates a decrease from 3393 annotated entries to 2762 between 2015 and 2025. The most abundant activities found were (i) MF: sodium/ion channel activity til 2015, then ‘toxin activity’ was most abundant; (ii) BP: ‘pathogenesis’ and ‘defense response’ til 2015, then ‘defense response’; and (iii) CC: consistently recover ‘extracellular region/space’ as most abundant across time (Fig. S4).

### Functional clustering of Tox-Prot entries as an additional information level

Protein family categorization relies on sequence similarities, conserved motifs, evolutionary relationships, and structural information [13]. However, frequent revisions in venom protein family nomenclature (e.g. the Huwenotoxin-1 family renamed Neurotoxin 10 (Hwtx-1)) and the ongoing aggregation or subdivision of venom protein groupings, such as spider neurotoxins and 3FTxs [24,25], suggest the need for a more function-oriented classification approach that complements sequence-based methods.

ProtSpace, a recently developed visualization tool, enables protein clustering based on protein language model (pLM) embeddings rather than direct sequence or structural similarity [20]. These embeddings, generated by models trained on millions of protein sequences, capture complex functional and structural relationships encoded in protein sequences [26]. By reducing the dimensionality of these high-dimensional representations, ProtSpace enables the exploration of functional relationships between proteins that may not be immediately apparent from sequence alignment alone. For instance, the original ProtSpace study demonstrated that venom proteins from marine cone snails and terrestrial spiders exhibit closer functional relationships in embedding space than traditional nomenclature would suggest [20].

**Figure 7:**
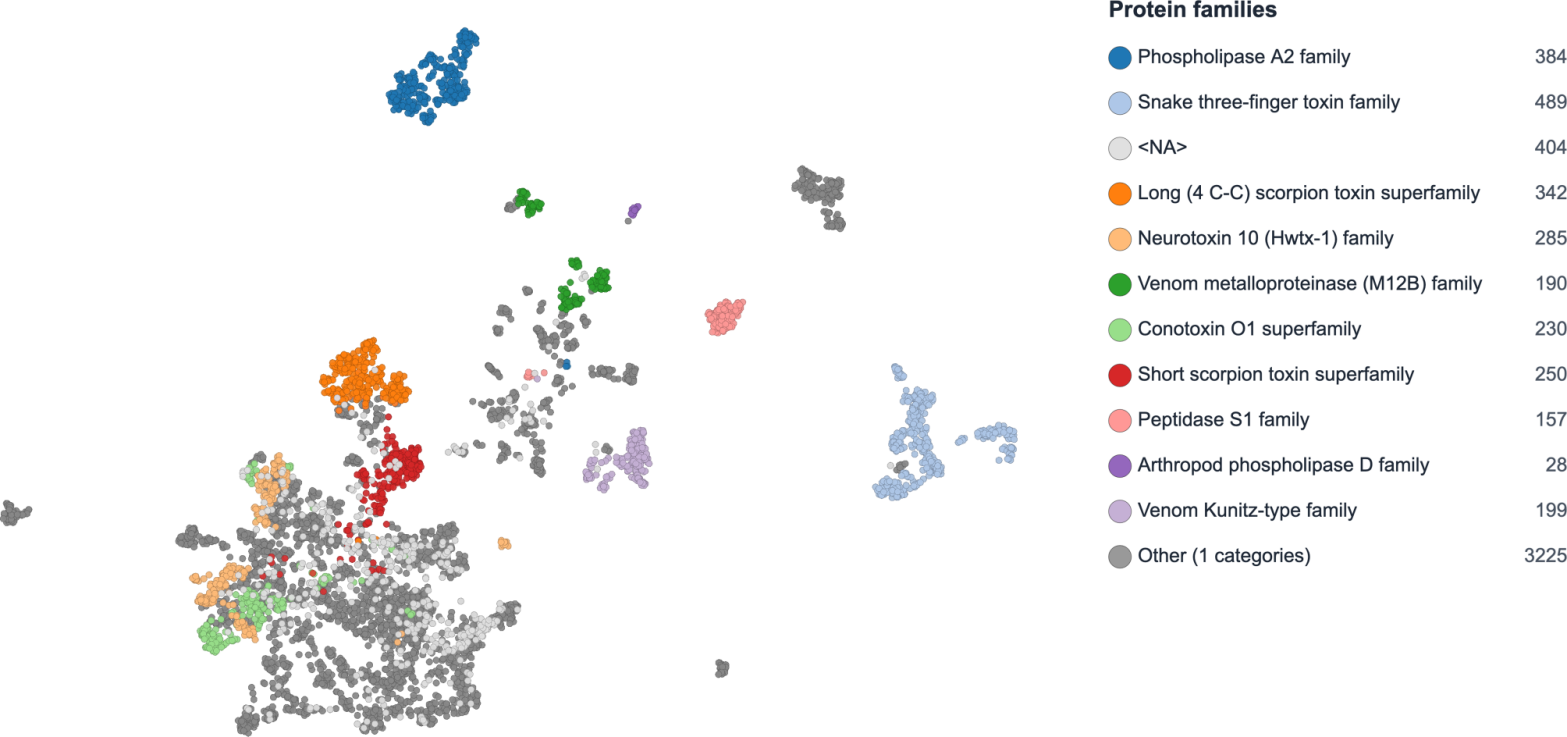
Functional clustering of the 10 most abundant venom protein families in Tox-Prot 2025 reveals distinct separation between short peptide toxins and large enzymatic families. Two-dimensional UMAP [21] projection of ProtT5 embeddings [19], where each point represents a venom protein coloured by protein family assignment. Large enzymatic families (Phospholipase A2, Venom metalloproteinase, and Peptidase S1) form well-separated clusters, while short peptide toxin families (scorpion toxins, conotoxins, and neurotoxins) occupy a more interconnected region. Entries with unassigned family annotation (<NA>) and minor families (Other) are shown in light and dark grey, respectively. Signal peptides were removed based on UniProtKB annotations, and fragment sequences were excluded prior to embedding generation, yielding improved cluster separation (silhouette score 0.474; see Fig. S5 for variant comparison). Protein families are colour-coded as in Fig. 3.

We applied ProtSpace to the 10 most abundant protein families in the 2025 Tox-Prot dataset. To optimize clustering quality, we compared five sequence processing variants: full-length sequences including signal peptide, sequences with signal peptides removed based on UniProtKB annotations, sequences with signal peptides removed excluding fragment entries, signal peptides and propeptides removed, and signal peptides and propeptides removed excluding fragments. Silhouette score analysis showed that signal peptide removal substantially improves cluster separation, while additional propeptide removal slightly decreases clustering quality (0.262, 0.397, 0.474, 0.382, and 0.401, respectively; Fig. S5). Removing propeptides reduces information available to the embedding model, explaining the decrease in clustering quality. We selected the signal peptide removed, fragment-free dataset for visualization (Fig. 7), as it achieved the highest silhouette score. The analysis revealed distinct clustering patterns: large enzymatic venom protein families such as PLA2, Venom metalloproteinase (M12B), and Peptidase S1 formed well-separated clusters, while predominantly short peptide toxin families—including scorpion toxins, conotoxins, and neurotoxins—occupied a more interconnected region of the embedding space, reflecting shared functional characteristics across these families. These observations support the hypothesis that evolutionary and taxonomic context alone may be insufficient for classifying animal venom proteins, emphasizing the value of complementary function-based classification approaches.

## Discussion

Our comparative analysis of Tox-Prot entries over two decades (2005–25) highlights two phases of database development: rapid initial growth, followed by a consolidation phase, marked by slower growth and greater data diversification. During this period the venom-related dataset grew by overall 438%, with a marked increase in Araneae and Neogastropoda entries around 2010, potentially indicating the incorporation of Arachnoserver and ConoServer. The persistent dominance of the same four major taxonomic orders (Squamata, Araneae, Neogastropoda, and Scorpiones), even in alternating ranking across time, likely reflects a historical emphasis on medically relevant or more easily sampled taxa [e.g. *27*]. The addition of new and rare taxonomic groups, including polychaetes, remipedes, ants, and different fish species, reflects that recent technological advances in genomics, transcriptomics, and proteomics are gradually enabling researchers to explore smaller and more elusive venom systems [e.g. 28].

The temporary disappearance of taxonomic orders across our study timeline may reflect ongoing controverse debates over the definition of venom systems, their description in novel species, and the distinction between venoms, poisons, and toxins. This particularly applies to the debate on poisonous versus venomous frogs [29] and the classification of saliva as venomous when exhibiting paralytic and liquefying activity as described in assassin bugs [30]. This controversy extends further to the observation that the phyla Cnidaria, Nemertea, and Porifera are fully excluded from the dataset when restricting the query to venom-related entries, without the addition of toxins lacking venom tissue specificity (Fig. S1). In the case of Cnidaria (jellyfish, sea anemones, and corals), this is striking as they are well accepted as ancient venomous taxa [31], but the expression of venoms in specialized cells, i.e. the nematocytes, instead of complex tissues or glands, may be the main factor for their exclusion from the venom-related Tox-Prot dataset.

The representation of terrestrial and marine venomous species across Tox-Prot over time indicates that marine venomous species account for 16%–20% of the taxonomic diversity. Considering the current estimate of venomous species across habitats, i.e. 12% marine of c. 226 000 venomous species [23], this would translate into an overrepresentation of marine taxa in Tox-Prot. However, upon closer inspection of the distribution of marine species in our datasets, we observe that between 2005 and 2025, 63%–85% of these species are Neogastropoda, which, together with the remaining molluscs, account for only 32% of the estimated natural marine venomous species diversity [23]. For better comparability, we further revised the relative abundance of Neogastropoda among marine venomous species in the database compared to current species diversity estimates, when including the complete Tox-Prot database (i.e. venom- and toxin-related entries). This leads to an increase in marine species up to c. 26% and a relative abundance of Neogastropoda among them of c. 39%–56%, thus, still far beyond the natural representation of the marine realm. The opposite underrepresentation of Hymenoptera in the terrestrial realm in comparison to snakes and scorpions, and especially in the broader context of the three-quarter terrestrial species diversity being insects [23], indicates that besides habitat-related factors other limitations hamper the assessment of venoms and potentially affecting their representation in the database. These factors comprise the accessibility of the venomous species, the medical relevance—as historically those species most harmful for humans have been more deeply studied—and the amount and purity of their venoms. The latter is often limited by the nature of venom-expressing tissue or gland and its surrounding tissues, which can challenge the venom extraction by adding contaminants such as mucus, as often the case in marine animals [e.g. 7, 32].

Our findings indicate PLA2s, 3FTxs, and Long 4(C-C) scorpion toxins are predominant venom protein families in Tox-Prot over the study period of 2005–25. These protein families have been shown to effectively disrupt the physiological functions of target organisms, supporting their dominance in potent, fast-acting venom cocktails. 3FTxs are so named due to their common folding scaffold of three β-stranded loops around a central core stabilized by four disulfide bonds. Despite high structural similarity, 3FTxs exhibit a wide range of activities, including neurotoxicity and pain induction, as well as effects on blood coagulation and cardiotoxicity [33, 34]. In comparison, the PLA2s found mostly in snake and insect venoms are secretory PLA2s classified in groups based on their conserved structure and disulfide scaffold, as well as the presence of a calcium-binding loop. These catalytic polypeptides hydrolyze membrane phospholipids and contribute to inflammatory, analgesic, and cardiovascular effects, often related to the envenomation process [35, 36]. Long 4(C-C) scorpion toxins are neurotoxic compounds divided into two main subfamilies: α-toxins and β-toxins. They inhibit voltage-gated sodium channels in nerves and muscles, thereby prolonging channel inactivation and inducing pain and cardiovascular impairment [37]. The potent and damaging effect of these three venom compounds may explain their predominance across taxa in the Tox-Prot database.

Another venom family found to be widely distributed across taxa and in both terrestrial and marine habitats was the Venom Kunitz-type family. Kunitz-type proteins are the best characterized serine protease inhibitors known to date. They are 50–60 aa long and composed nearly entirely of the Kunitz-domain stabilized by a conserved three-disulfide bridge motif. Kunitz-like proteins are known to impact mostly blood coagulation, inflammatory processes, and fibrinolysis [38]. Like 3FTXs and Long 4(C-C)s, some Venom Kunitz-type proteins can have neurotoxic effects, specifically by targeting voltage-gated calcium (Ca^2+^) and potassium (K^+^) ion channels. Their wide appearance across the taxonomic landscape of venomous species might be related to the evolutionary origin and biological significance of protease inhibitors in major physiological processes, including the cell cycle, hemostasis, and apoptosis [38].

The abundance of peptides and small proteins (26–75 aa) and the strong record of disulfide bridges as the most prominent PTMs align with the numerous studies highlighting the importance of identifying the correct disulfide bond scaffold for folding and bioactivity, especially in the context of recombinant expression and drug development [39, 40]. More information is required on the relevance for protein structure, functionality, and target interaction of other PTMs found annotated in the database, such as the second and third most abundant, amidation and glycosylation. C-terminal amidation is, for example, highly common in cone snail [41] and wasp venom peptides [42], and its presence is crucial for their bioactivity and structural integrity with potency severely reduced when absent [43]. Traditionally, glycosylation of proteins (glycoproteins) is involved in physiological processes such as protein quality control, subcellular localization, cell–cell interactions, maintenance of protein structure and function, and improvement of protein folding and stability [44]. In venom proteins, especially in snake venom serine proteases, glycosylation has been shown to contribute significantly to the recorded magnitude of activity in comparison to the deglycosylated counterpart [44].

The embedding-based clustering of venom protein families (Fig. 7) reveals a fundamental dichotomy in venom protein architecture: large enzymatic families such as PLA2, Venom metalloproteinase (M12B), and Peptidase S1 occupy distinct, well-separated regions, while short disulfide-rich peptide toxins—including scorpion toxins, conotoxins, and neurotoxins—form a more interconnected cluster despite their diverse taxonomic origins. This pattern suggests that short peptide toxins from phylogenetically distant taxa converge on similar structural scaffolds for targeting ion channels and receptors, consistent with the shared pharmacological profiles observed across terrestrial and marine venomous species. Such functional convergence, captured here through protein language model embeddings without relying on direct sequence alignment, reinforces the limitations of purely sequence-based nomenclature and supports complementary function-oriented classification approaches. The growing potential of machine learning for predicting functional relationships among the enormous diversity of estimated venom biomolecules [45] is further reflected in recent tool developments such as the Molecular Arms Race Classifier (MARC) [46], ConoDictor [47], and TOXIFY [48].

Tox-Prot currently represents the most centralized data hub for the venomics field. By its very nature, no curated database can comprehensively capture all research activity within a field, owing to inherent constraints in data accessibility, curation workflows, and selection criteria. However, even Tox-Prot does not include the entirety of venom research, our results are in strong accordance with taxonomic, protein family, and PTM patterns recorded in literature [e.g. 3, 28, 34]. We therefore consider our database-based evaluation to be indicative rather than comprehensive, yet sufficiently robust to highlight key trends in venom research in the last two decades such as the continued growth of venom data, increasing taxonomic diversification, the predominance of certain protein families likely reflecting taxonomic sampling bias, and the central role of PTMs, particularly disulfide bridges and amidation.

The Tox-Prot database evaluation further indicates limitations and hurdles in venomics: (i) Incomplete sequences: One sixth of the entries are fragments, most of them incomplete mature protein sequences, leading to missing information, low comparability, misidentification, and reduced usability for synthesis and bioactivity profiling. (ii) Nomenclature: The large number of apparently ‘new’ protein families is often related to renaming driven by database growth and increasing diversity of sequence, structural, and bioactivity information. This supports earlier calls for a rationalized nomenclature, as advances in venom research have enabled the rapid identification of peptides and proteins in the absence of unified naming conventions [49–51]. Historically, these molecules have been named based on species of origin, structure, activity, or purification method [49, 50]. Growing taxonomic and chemical diversity highlights the need for standardized nomenclature and a reassessment of protein family classification criteria. (iii) Taxonomic disparity: Given the biased representation towards Squamata, scorpions, and cone snails in Tox-Prot, increased efforts are needed to balance the full diversity of venomous taxa. Further, revision of the annotation or definition of proteins and peptides from Cnidaria, Nemertea, and Porifera as venom system-related should be considered, as well as the better data representation of insects. To better understand the origin, evolutionary adaptations, bioactivity, and implications for drug discovery and development, this gap needs to be addressed. (iv) The need for a unifying database that integrates all venomics data in a multimodal approach, connecting all metadata available across taxa and disciplines, including genomics, proteomics, transcriptomics, morphology, and structure and function. The VenomsBase project is the most recent example of bundled efforts to address these challenges and build a novel datahub for animal venomics [9].

The findings in the present study indicate that the venom research field is vibrant, continuously expanding, and would benefit from a centralized knowledge base to address taxonomic gaps and nomenclature inconsistencies. The patterns drawn herein from our analysis of Tox-Prot venom data add a molecular perspective on venom composition beyond taxonomic relationships, by identifying parallels in protein families across terrestrial and marine habitats, and by highlighting the importance of functional clustering of venom compounds. We strongly encourage following up on this functional protein family relationship in a species ecology context to better understand potential triggers for the evolutionary origin and adaptation of venom systems.

## Supplementary Material

baag032_Supplemental_Files

## Data Availability

The Tox-Prot database is publicly available. The raw Tox-Prot downloaded data presented in this study are available in Supplementary Data S1.
