# Supplementary figures and images for: Quantitative analysis of growth and diversification in venom data using database metrics
Source: Database (Oxford). 2026 May 30;2026:baag032. doi: 10.1093/database/baag032 (PMC13221835; doi:10.1093/database/baag032)

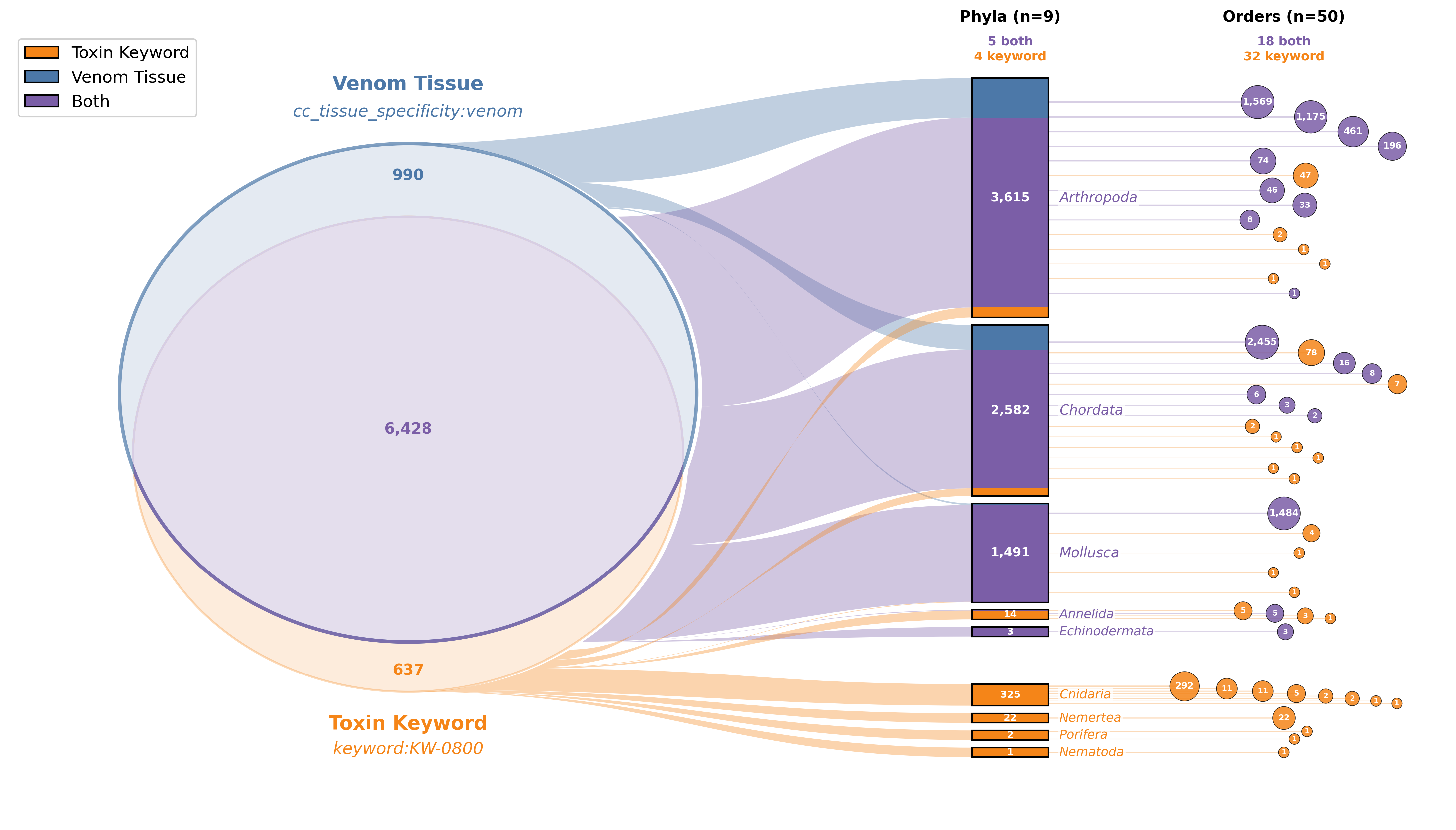

Supplement: baag032_Supplemental_Files [file baag032_supplemental_files.zip › SupplementaryFile_FigS1_DefinitionQueryTerms.png]

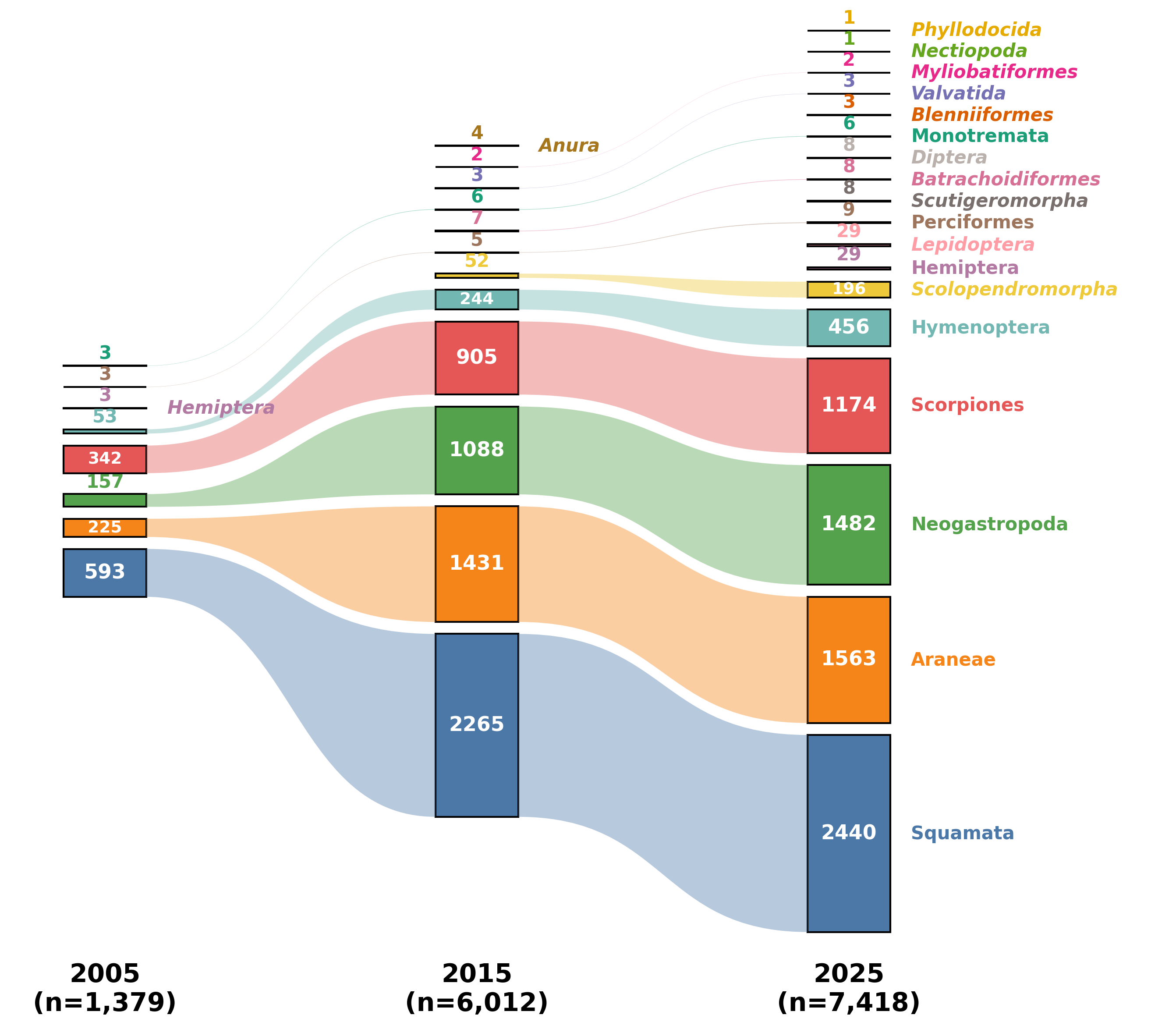

Supplement: baag032_Supplemental_Files [file baag032_supplemental_files.zip › SupplementaryFile_FigS2_TaxaNewcomersOrders_alluvial.png]

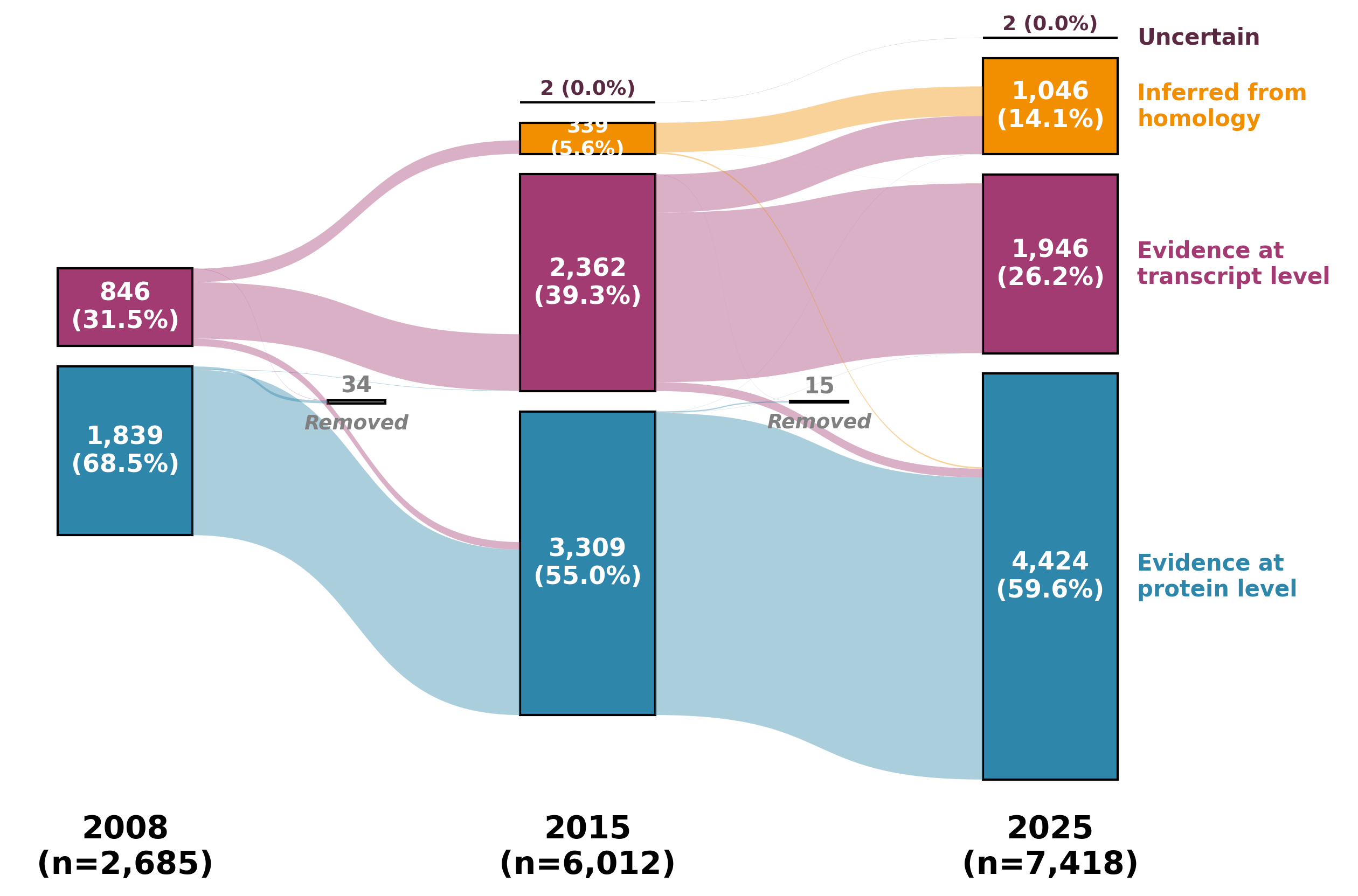

Supplement: baag032_Supplemental_Files [file baag032_supplemental_files.zip › SupplementaryFile_FigS3_protein_evidence_sankey.png]

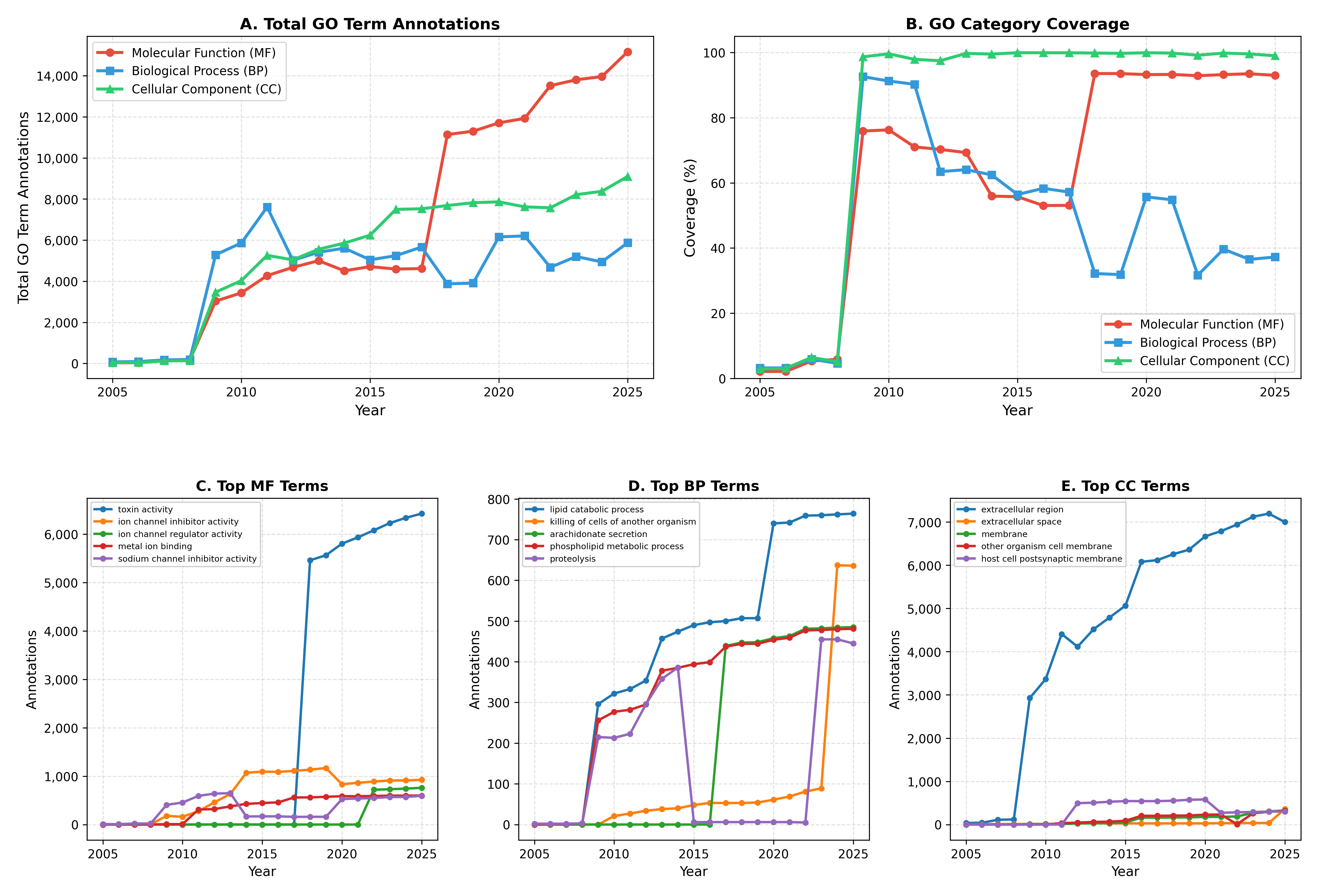

Supplement: baag032_Supplemental_Files [file baag032_supplemental_files.zip › SupplementaryFile_FigS4_FunctAnnotation.png]

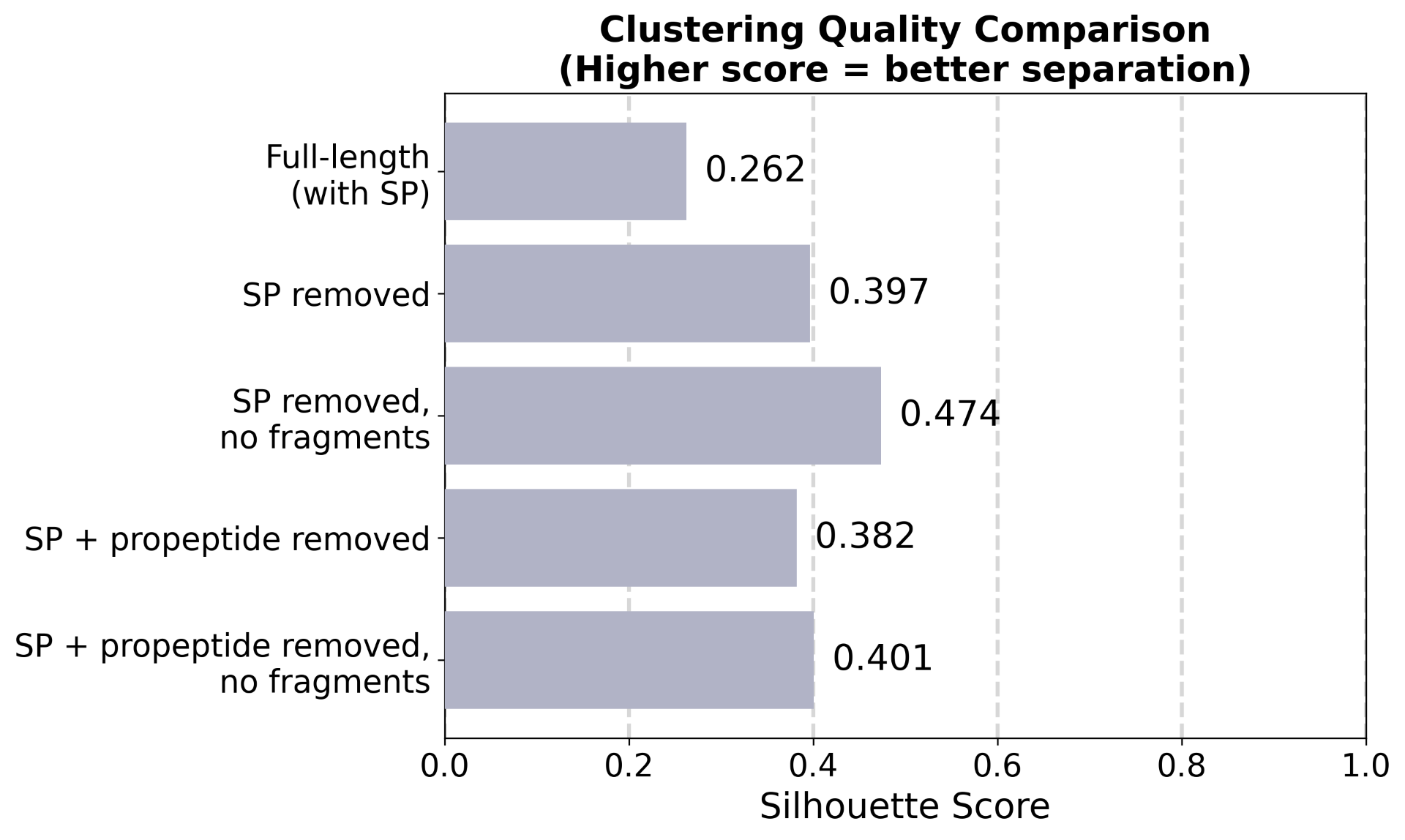

Supplement: baag032_Supplemental_Files [file baag032_supplemental_files.zip › SupplementaryFile_FigS5_SilhouetteAnalysis.png]
